# DNA Damage in CD133-Positive Cells in Barrett's Esophagus and Esophageal Adenocarcinoma

**DOI:** 10.1155/2016/7937814

**Published:** 2016-03-10

**Authors:** Raynoo Thanan, Ning Ma, Yusuke Hiraku, Katsunori Iijima, Tomoyuki Koike, Tooru Shimosegawa, Mariko Murata, Shosuke Kawanishi

**Affiliations:** ^1^Department of Biochemistry, Faculty of Medicine, Khon Kaen University, Khon Kaen 40002, Thailand; ^2^Faculty of Nursing Science, Suzuka University of Medical Science, Suzuka, Mie 513-8670, Japan; ^3^Department of Environmental and Molecular Medicine, Mie University Graduate School of Medicine, Tsu, Mie 514-8507, Japan; ^4^Division of Gastroenterology, Tohoku University Hospital, Sendai 980-8574, Japan; ^5^Faculty of Pharmaceutical Sciences, Suzuka University of Medical Science, Suzuka, Mie 513-8670, Japan

## Abstract

Barrett's esophagus (BE) caused by gastroesophageal reflux is a major risk factor of Barrett's esophageal adenocarcinoma (BEA), an inflammation-related cancer. Chronic inflammation and following tissue damage may activate progenitor cells under reactive oxygen/nitrogen species-rich environment. We previously reported the formation of oxidative/nitrative stress-mediated mutagenic DNA lesions, 8-oxo-7,8-dihydro-2′-deoxyguanosine (8-oxodG) and 8-nitroguanine, in columnar epithelial cells of BE tissues and cancer cells of BEA tissues. We investigated the mechanisms of BEA development in relation to oxidative/nitrative DNA damage and stem cell hypothesis. We examined 8-nitroguanine and 8-oxodG formation and the expression of stem cell marker (CD133) in biopsy specimens of patients with BE and BEA by immunohistochemical analysis in comparison with those of normal subjects. CD133 was detected at apical surface of columnar epithelial cells of BE and BEA tissues, and the cytoplasm and cell membrane of cancer cells in BEA tissues. DNA lesions and CD133 were colocalized in columnar epithelial cells and cancer cells. Their relative staining intensities in these tissues were significantly higher than those in normal subjects. Our results suggest that BE columnar epithelial cells with CD133 expression in apical surface undergo inflammation-mediated DNA damage, and mutated cells acquire the property of cancer stem cells with cytoplasmic CD133 expression.

## 1. Introduction

Chronic inflammation during gastroesophageal acid reflux disease (GERD) is an important risk factor of Barrett's esophagus (BE) and esophageal carcinogenesis [1, 2]. BE is defined as the presence of a metaplastic columnar-lined esophagus induced by GERD. BE patients have 30–40-time larger risk of Barrett's esophageal adenocarcinoma (BEA) [3]. Mutations of p53 are clearly involved in the pathogenesis of BEA, and the fact that the mutations were detected in premalignant Barrett's epithelium supports the hypothesis that p53 mutations may be a useful marker for patients at increased risk for development of invasive cancer [4]. Reactive oxygen species (ROS) and reactive nitrogen species (RNS) are generated during inflammation and considered to contribute to inflammation-mediated carcinogenesis [5–7]. ROS and RNS can induce the formation of 8-oxo-7,8-dihydro-2′-deoxygaunosine (8-oxodG) and 8-nitroguanine, the markers of oxidative and nitrative DNA damage, respectively. Production of nitric oxide (NO) by inducible nitric oxide synthase (iNOS) and superoxide radical anion (O_2_^∙−^) by NAD(P)H oxidase contributes to peroxynitrite (ONOO^−^) generation to cause 8-nitroguanine formation [8]. Abundant amount of NO has been found in the human gastroesophageal junction, and NO could diffuse into the adjacent epithelium at cytotoxic levels resulting in the pathogenesis of GERD spectrum [9]. Moreover, overexpression of iNOS and its transcriptional factor (NF*κ*B) was detected in the order of BEA > BE > normal esophagus tissues and the suppression of Mn-SOD expression was also found in BE and BEA tissues [10]. These molecular events contribute to generation of ONOO^−^, resulting in the formation of DNA lesions [10]. Recently, oxidative DNA damage was also found to be associated with genetic instability via telomeric dysfunction, leading to p53 mutation and BEA tumorigenesis [11]. Therefore, oxidative and nitrative stress has been shown to increase during the development from BE to BEA through the induction of ROS- and RNS-generating enzymes, leading to an increase in DNA lesions, which contribute to mutations and genetic instability. However, the molecular mechanism of carcinogenesis has not fully been clarified.

Accumulating evidence in recent years strongly indicates the existence of cancer stem cells in tumors of a wide variety of organs, particularly in inflammation-associated cancers [12]. Inflammation-associated tissue injury may activate stem (progenitor) cells, and multiple mutagenic and epigenetic changes are accumulated in these cells under such conditions [13]. We have recently reported that oxidative and nitrative DNA damage occurred in cells positive for stem cell markers in tissues of parasite-induced urinary bladder cancer [14] and intrahepatic cholangiocarcinoma, which are typical inflammation-related cancers [15]. BE develops to intestinal-like structure for acid resistance during chronic GERD. It is suggested that BE is differentiated from adult stem cell lining at the basal layer of esophageal epithelium and bone marrow stem cells [16–18]. CD133 is a transmembrane glycoprotein expressed in progenitor cells during differentiation and associated with cancer stem cells in several solid tumors [19–23]. CD133 has the potential to differentiate benign tumors to malignant tumors in the tissues of Barrett's esophagus [24]. These findings raise the possibility that CD133 could be used as a cancer stem cell marker related to oxidative and nitrative stress in BEA. Therefore, we examined the formation of inflammation-related DNA lesions (8-nitroguanine and 8-oxodG) and a stem cell marker (CD133) in biopsy specimens of BEA patients in comparison with those of normal esophagus and BE tissues for understanding the mechanisms of GERD-induced esophageal carcinogenesis.

## 2. Materials and Methods

### 2.1. Human Subjects

All tissues used in this study were obtained from endoscopic biopsies or endoscopic mucosal resections from patients at Tohoku University hospital as described previously [10]. Biopsy specimens were obtained from 19 BE patients (14 males and 5 females, mean ± SD, 63.6 ± 12.4 years), 11 BEA patients (10 males and 1 female, 66.5 ± 12.8 years), and 7 subjects with normal esophagus (4 males and 3 females, 58.4 ± 6.2 years). These specimens were formalin-fixed and embedded in paraffin. Among BEA patients, 10 cases were identified as stage I (well-differentiated adenocarcinoma) and 1 case was identified as stage II (moderately differentiated adenocarcinoma). In BE patients, only those with histological confirmation of specialized intestinal metaplasia and three or more centimeters of macroscopic Barrett's epithelium were included. BEA was defined by adenocarcinoma predominantly involving the tubular distal esophagus and histological evidence of adjacent Barrett's epithelium. In addition, subjects with macroscopically and histologically normal esophagus attending endoscopy for routine diagnostic procedure were recruited as controls. No participants administered acid suppression therapies, such as proton pump inhibitor or H2-blocker before endoscopic procedure. This study was approved by Tohoku University Hospital Ethics Committee (number 2003-149) and written informed consent was obtained from all subjects.

### 2.2. Immunohistochemical Study

Double or single fluorescent immunohistochemistry was performed to examine the colocalization of CD133, 8-nitroguanine, and 8-oxodG as described previously [25]. Paraffin sections were incubated with the primary antibodies [rabbit polyclonal anti-CD133 antibody (1 : 500, Abcam, Cambridge, UK), rabbit polyclonal anti-8-nitroguanine antibody (1 *μ*g/mL) produced by our group [25, 26], and mouse monoclonal anti-8-oxodG antibody (1 : 200, Japan Institute for the Control of Aging, Fukuroi, Japan)] overnight at room temperature. The sections were next incubated with fluorescent secondary antibodies (Alexa 488-labeled goat anti-mouse IgG and/or Alexa 594-labeled goat anti-rabbit IgG antibodies, 1 : 400 each, Molecular Probes Inc., Eugene, Oregon, USA) for 3 h at room temperature. Finally, the nuclei were stained by 4′-6-diamidino-2-phenylindole (DAPI) and the sections were examined with a fluorescence microscope (LX70, Olympus, Tokyo, Japan) or a laser scanning confocal microscope (Fluoview FV1000-D, Olympus) [10].

### 2.3. Immunohistochemical Grading

We defined immunohistochemical grading (IHC grading) based on the intensity and frequency derived from the staining results in normal mucosal, columnar, and cancer cells of normal esophageal, BE, and BEA tissues, respectively, according to the method described in our previous reports [10, 14, 27]. The staining intensity was scored as negative (0), weak (+1), moderate (+2), or strong (+3). The frequency of positive cells in a section was scored as negative (0), less than 25% (+1), 25–50% (+2), 51–75% (+3), or more than 75% (+4). An IHC score was assigned by multiplying the intensity score by the frequency score. IHC grading was assigned by an IHC score as follows: −, negative expression (0); +, weak expression (1–3); ++, moderate expression (4–6); +++, high expression (7–9); or ++++, very high expression (10–12). The IHC grading scores of each sample were approved by the expert pathologist.

### 2.4. Statistic Analysis

The statistically significant difference among normal, BE, and BEA groups was analyzed by chi-square test. Spearman rank correlation coefficients were calculated between DNA damage and CD133 staining levels. *P* < 0.05 was considered to be statistically significant.

## 3. Results

### 3.1. Subcellular Expression of CD133 in Normal Esophageal, BE, and BEA Tissues

**Figure 1:**
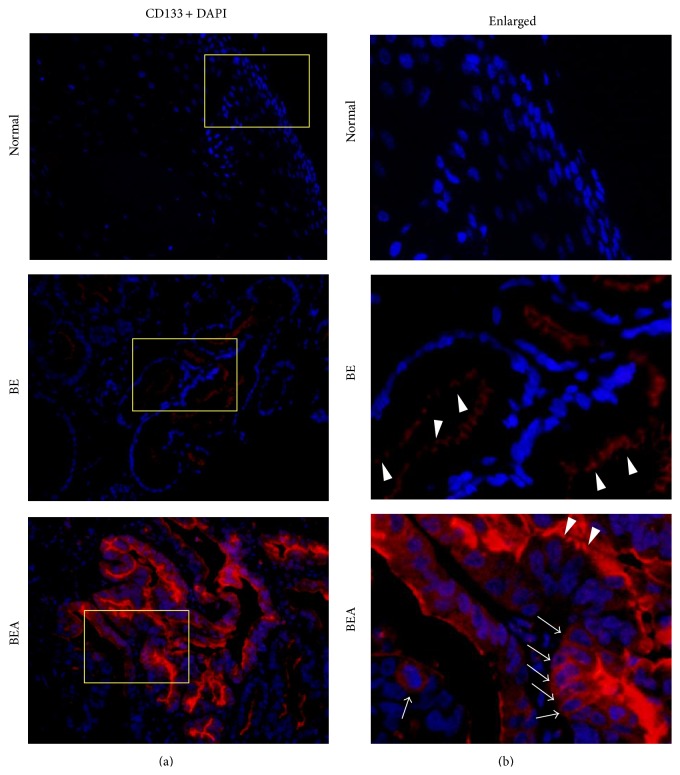
CD133 expression in normal esophageal (Normal), Barrett's esophagus (BE), and Barrett's adenocarcinoma (BEA) tissues. CD133 expression (red) was examined by immunofluorescence technique. DAPI (blue) was used for nucleic counterstaining. The original magnification is ×200 (a). (b) represent enlarged pictures of the yellow boxes in (a). Arrowheads indicate CD133 expression at apical surface. Arrows indicate cell membrane and cytoplasmic CD133 staining.

Figure 1 shows the localization of CD133 in normal esophageal, BE, and BEA tissues examined by fluorescent immunohistochemistry. CD133 was not stained in normal esophageal tissues and its expression was significantly increased in BE and BEA tissues. Interestingly, CD133 was weakly detected at apical surface of metaplastic columnar cells in BE tissues and highly detected in cancer cells in BEA tissues (Figure 1, enlarged; arrowheads). Interestingly, cell membrane and cytoplasmic CD133 staining was detected only in cancer cells of BEA tissues (Figure 1, enlarged; arrows).

Apical surface staining of CD133 was significantly higher in BE and BEA subjects compared with normal subjects (Table 1, *P* = 0.005 and *P* = 0.027, resp.), and there was significant difference between BE and BEA (*P* = 0.038). CD133 staining in the cytoplasm and cell membrane was observed in BEA tissues alone and showed a significant increase compared with BE tissues (*P* = 0.001). There was a nonsignificant difference in CD133 staining between normal and BEA tissues (*P* = 0.063), probably because of small sample size.

### 3.2. Inflammation-Related DNA Lesion in Normal Esophageal, BE, and BEA Tissues

**Figure 2:**
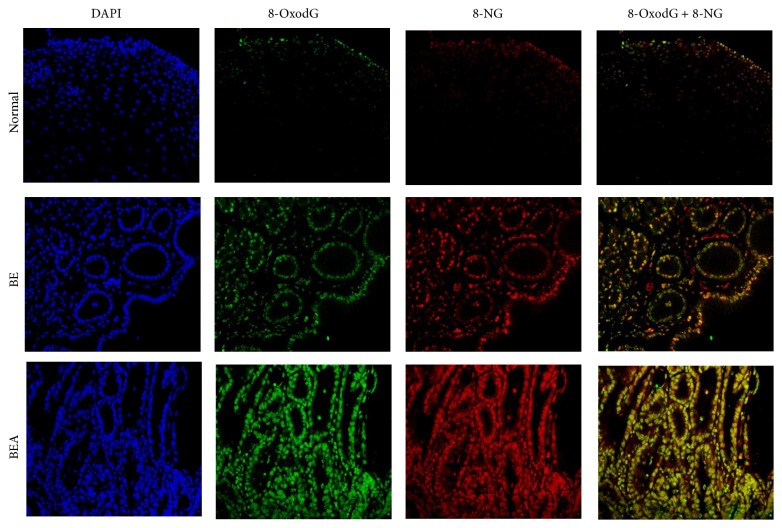
Double immunofluorescence staining of mutagenic DNA lesions in normal esophageal (Normal), Barrett's esophageal (BE), and Barrett's esophageal adenocarcinoma (BEA) tissues. Nucleus was stained in blue (DAPI). 8-OxodG was stained in green and 8-nitroguanine (8-NG) was stained in red. The original magnification is ×200.

Figure 2 shows the localization of 8-oxodG and 8-nitroguanine in normal esophageal, BE, and BEA tissues examined by fluorescent immunohistochemistry. 8-OxodG and 8-nitroguanine were weakly formed in nucleus of normal esophageal tissues, whereas they were highly formed in the nucleus of BE and BEA. Cells positive for both 8-oxodG and 8-nitroguanine were significantly increased in BE and BEA tissues compared with normal subjects (*P* < 0.001 and *P* = 0.001, resp.), and tended to increase in BEA subjects compared with BE subjects (*P* < 0.088) as shown in Table 1. Our preliminary study indicated that phosphorylated H2AX (*γ*-H2AX), as another DNA damage marker, was observed in the nucleus of BEA tissues (Supplementary Figure  1) (see Supplementary Material available online at http://dx.doi.org/10.1155/2016/7937814).

### 3.3. Detection of DNA Damage in CD133-Positive Cells of BE and BEA Tissues

**Figure 3:**
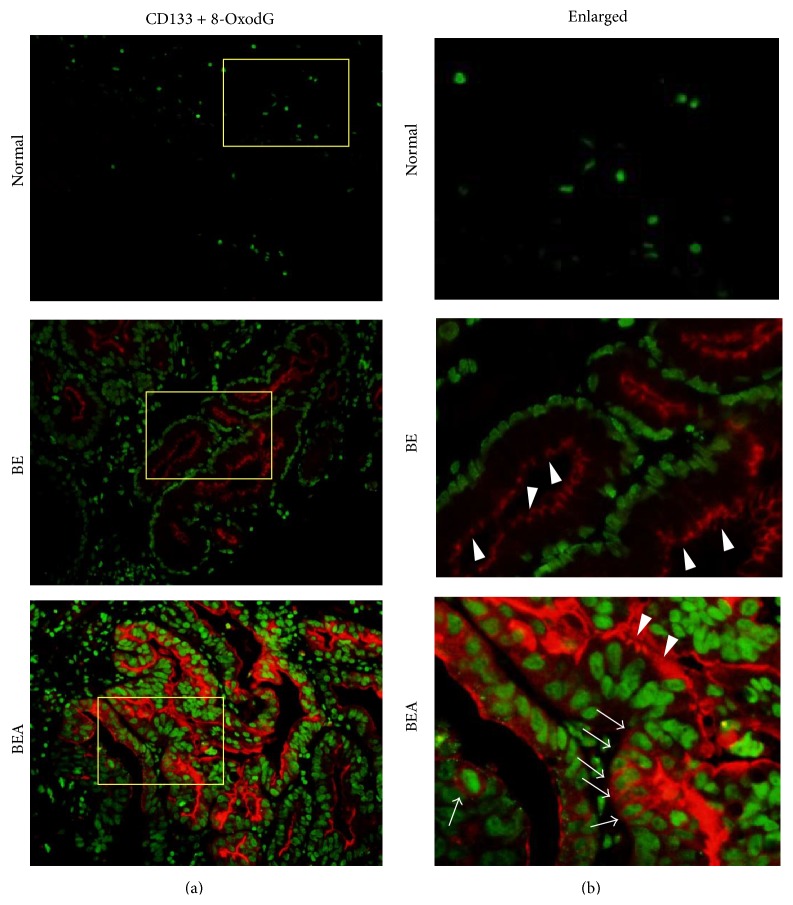
Double immunofluorescence staining of CD133 and 8-oxodG in normal esophageal (Normal), Barrett's esophageal (BE), and Barrett's esophageal adenocarcinoma (BEA) tissues. CD133 and 8-oxodG were stained in red and green, respectively. The original magnification is ×200 (a). (b) represent enlarged pictures of the yellow boxes in the (a). Arrowheads indicate CD133 expression in apical surface. Arrows indicate cell membrane and cytoplasmic CD133 staining.

The oxidative DNA damage marker (8-oxodG) was found in CD133-positive cells in BE and BEA tissues as shown in Figure 3. Moreover, the DNA lesion was also formed in cell membrane and cytoplasmic CD133-positive cells. Interestingly, the intensity of DNA damage was significantly correlated with CD133 expression at the cytoplasm and cell membrane (*r* = 0.405, *P* = 0.013 by Spearman rank correlation), whereas no correlation was found with CD133 expression at columnar apical surface.

## 4. Discussion

We showed here that DNA lesions and the stem cell marker CD133 were colocalized in columnar gland cells in BE tissues and cancer cells in BEA tissues. 8-Nitroguanine and 8-oxodG are mutagenic lesions leading to point mutation (G to T transversion) [28]. These DNA lesions could be detected in the nucleus of cancer stem-like cells in cholangiocarcinoma and bladder cancers, which may be involved in inflammation-driven carcinogenesis [14, 15, 27]. 8-OxodG and 8-nitroguanine were formed in the nucleus of several inflammation-related cancers such as liver fluke-associated cholangiocarcinoma [29]. Our previous studies confirmed the formation of 8-oxodG in the livers of liver fluke-infected hamster models [30], and the increase in cancer cells of human cholangiocarcinoma tissues [15] was detected by both immunohistochemistry and HPLC coupled with electrochemical detector (ECD). Both techniques showed similar results, and therefore, we used immunohistochemical method in the present study, because of limited amounts of biopsy samples. The amount of 8-oxodG excreted in urine, which was measured by HPLC-ECD, was significantly increased in parasite-infected subjects and cholangiocarcinoma patients compared with healthy control subjects [31]. Recently, ELISA technique indicated that urinary 8-oxodG levels were correlated with hepatobiliary pathology of the liver fluke infection, which is associated with cholangiocarcinogenesis [32]. Accumulated evidence suggested that 8-oxodG and 8-nitroguanine could be used as the potential inflammation-related DNA damage markers. BE and BEA are inflammation-related diseases induced by gastric reflux components [1]. Our previous study reported that oxidative stress plays an important role in BE and BEA development [10]. Consequently, we have hypothesized that the formation of inflammation-related DNA lesions in progenitor-like cells in BE is involved in BEA carcinogenesis. This process could be explained by the accumulation of mutation in progenitor cells, leading to the acquisition of the property of cancer stem cells.

CD133 was originally identified as a transmembrane glycoprotein in normal hematopoietic stem and progenitor cells that participated in proliferation, self-renewal, and multilineage differentiation [33]. CD133 has been characterized as a marker for tumor-initiating cells in gastrointestinal tract system including colon, gastric, pancreatic, and liver cancer [15–19, 26]. The present results indicated that CD133 expression was significantly increased in BE and BEA tissues compared with normal tissues, all of which were CD133-negative. CD133 was positive in cell membrane and the cytoplasm only in BEA tissues but not in BE tissues, and there was a significant difference between these groups as shown in Table 1. CD133 expression was observed in apical surface of columnar glands in BE and BEA tissues. Our results are supported by a recent study showing that apical CD133 expression was observed in dysplastic Barrett's esophagus and esophageal adenocarcinoma [24]. Some other immunohistochemical studies showed the significance of CD133 expression in esophageal cancer [33, 34]. The cytoplasmic localization of CD133 was also found in cholangiocarcinoma, which is also a cancer of epithelial cells [15]. In addition, CD133 was detected in apical membrane of epithelial cells of normal minor salivary glands in normal subjects and adenoid cystic carcinoma patients [35]. Moreover, it was expressed in luminal (apical) surface membrane of gland-forming cells [36]. Cytoplasmic CD133 overexpression might be a useful marker of prognosis of gastric cancer [37, 38]. Sasaki et al. also reported that cytoplasmic expression of CD133 was a significant risk factor for the overall survival and tumor stages III and IVA of hepatocellular carcinoma patients [39]. Recently, nuclear and cytoplasmic CD133 was also detected in nonsmall cell lung cancer tissues and correlated with poor prognosis [40]. From previous literatures and our results, it is speculated that CD133 expression in apical surface of epithelial cells means normal stem cell differentiation, whereas its expression in cell membrane and the cytoplasm is associated with the properties of cancer stem cells. Therefore, cytoplasmic CD133 expression could be a marker of cancer stem cells in BEA.

**Figure 4:**
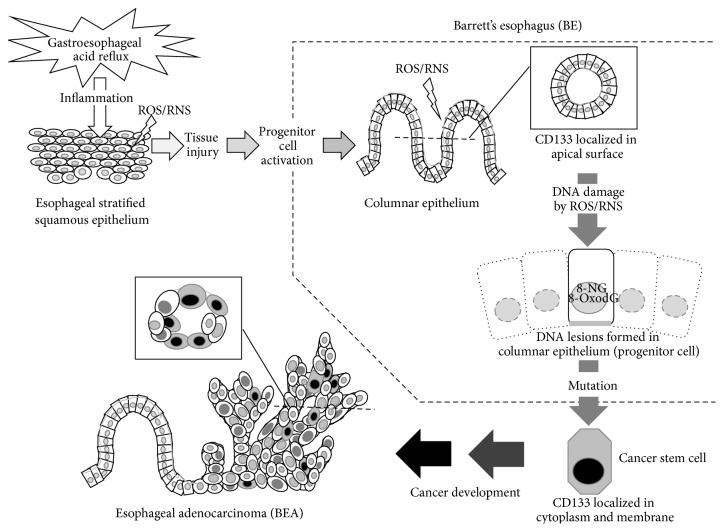
Proposed mechanism of Barrett's esophageal carcinogenesis (BEA) derived from Barrett's esophagus (BE). GERD induces inflammatory responses and tissue injury, which mediate intestinal dysplasia and CD133 expression in apical surface of columnar epithelial cells. Inflammatory responses also mediate DNA damage in these cells with progenitor-like properties, which may lead to accumulation of mutations. Under such conditions, the alteration in CD133 localization to cell membrane and the cytoplasm occurs, and the cells acquire the property of cancer stem cells, leading to BEA development.

The formation of mutagenic DNA lesions, including 8-nitroguanine and 8-oxodG, was significantly and positively correlated with each CD133 staining pattern of cells in BE and BEA tissues. The proposed mechanism of BE-derived esophageal carcinogenesis mediated by GERD is shown in Figure 4. GERD induces inflammatory responses and tissue injury, which mediate intestinal dysplasia and CD133 expression in apical surface of columnar epithelial cells. Inflammatory responses also mediate DNA damage in these cells with progenitor-like properties, which may lead to accumulation of mutations. Under such conditions, the alteration in CD133 localization to cell membrane and the cytoplasm occurs, and the cells acquire the property of cancer stem cells, leading to BEA development. This mechanism is supported by recent studies showing that chronic inflammation and following tissue damage may activate progenitor cells under ROS- and RNS-rich environment [14, 15, 27, 41]. In conclusion, oxidative and nitrative DNA lesions and differential CD133 localization would contribute to BE-derived carcinogenesis, and these molecules could be used as potential biomarkers to evaluate the risk of this disease.

## Supplementary Material

Immunohistochemical study of phosphorylated H2AX: Phosphorylated H2AX (γ-H2AX) was stained by using mouse monoclonal anti-phospho-H2AX antibody (clone JBW301, 4 μg/mL, Merck Milipore, Darmstadt, Germany) as a primary antibody. Paraffin sections were incubated with the primary antibodies overnight at room temperature. The sections were next incubated with fluorescent secondary antibody (1:400 Alexa 488-labeled goat anti-mouse IgG (Molecular Probes Inc., Eugene, Oregon, USA) for 3 h at room temperature. Finally, the nuclei were stained by 4'-6-diamidino-2-phenylindole (DAPI) and the sections were examined with a fluorescence microscope (LX70, Olympus, Tokyo, Japan) ora laser scanning confocal microscope (Fluoview FV1000-D, Olympus). For the negative control, we omitted the primary antibody and treated with the secondary antibody according to the procedure.

## Figures and Tables

**Table 1:** Immunoreactivity grading of CD133 among normal esophagus, Barrett's esophagus, and Barrett's adenocarcinoma tissues.

Factor	^$^Group	^#^IHC grade					^*∗*^ *P* value	
		−	+	++	+++	++++	vs. Normal	vs. BE
CD133 Apical surface	Normal	7	0	0	0	0		
	BE	4	5	4	6	0	*P* = 0.005	
	BEA	3	1	5	0	2	*P* = 0.027	*P* = 0.038

CD133 Cytoplasm and cell membrane	Normal	7	0	0	0	0		
	BE	19	0	0	0	0	*P* = 1.000	
	BEA	4	4	1	2	0	*P* = 0.063	*P* = 0.001

8-oxodG and 8-nitroguanine double staining	Normal	6	1	0	0	0		
	BE	0	4	7	7	1	*P* < 0.001	
	BEA	0	0	3	4	4	*P* = 0.001	*P* = 0.088

^#^An IHC grade was assigned to each specimen according to the degree of staining as described in Section 2. Normal = normal esophagus, BE = Barrett's esophagus, and BEA = Barrett's esophageal adenocarcinoma.

^*∗*^ *P* values were calculated by chi-square test (versus (vs.) Normal and versus BE).

^$^IHC grade was analyzed in normal mucosal, columnar, and cancer cells in normal esophageal, BE, and BEA tissues, respectively.

## References

[1] Abdel-Latif M. M., Duggan S., Reynolds J. V., Kelleher D. (2009). Inflammation and esophageal carcinogenesis. *Current Opinion in Pharmacology*.

[2] Poehlmann A., Kuester D., Malfertheiner P., Guenther T., Roessner A. (2012). Inflammation and Barrett's carcinogenesis. *Pathology Research and Practice*.

[3] Cameron A. J. (1997). Barrett's esophagus: does the incidence of adenocarcinoma matter?. *The American Journal of Gastroenterology*.

[4] Schneider P. M., Casson A. G., Levin B. (1996). Mutations of p53 in Barrett's esophagus and Barrett's cancer: a prospective study of ninety-eight cases. *Journal of Thoracic and Cardiovascular Surgery*.

[5] Coussens L. M., Werb Z. (2002). Inflammation and cancer. *Nature*.

[6] Kawanishi S., Hiraku Y. (2006). Oxidative and nitrative DNA damage as biomarker for carcinogenesis with special reference to inflammation. *Antioxidants and Redox Signaling*.

[7] Reuter S., Gupta S. C., Chaturvedi M. M., Aggarwal B. B. (2010). Oxidative stress, inflammation, and cancer: how are they linked?. *Free Radical Biology & Medicine*.

[8] Hiraku Y. (2010). Formation of 8-nitroguanine, a nitrative DNA lesion, in inflammation-related carcinogenesis and its significance. *Environmental Health and Preventive Medicine*.

[9] Iijima K., Shimosegawa T. (2014). Involvement of luminal nitric oxide in the pathogenesis of the gastroesophageal reflux disease spectrum. *Journal of Gastroenterology and Hepatology*.

[10] Thanan R., Ma N., Iijima K. (2012). Proton pump inhibitors suppress iNOS-dependent DNA damage in Barrett's esophagus by increasing Mn-SOD expression. *Biochemical and Biophysical Research Communications*.

[11] Cardin R., Piciocchi M., Tieppo C. (2013). Oxidative DNA damage in Barrett mucosa: correlation with telomeric dysfunction and p53 Mutation. *Annals of Surgical Oncology*.

[12] Takaishi S., Okumura T., Wang T. C. (2008). Gastric cancer stem cells. *Journal of Clinical Oncology*.

[13] Maitland N. J., Collins A. T. (2008). Inflammation as the primary aetiological agent of human prostate cancer: a stem cell connection?. *Journal of Cellular Biochemistry*.

[14] Ma N., Thanan R., Kobayashi H. (2011). Nitrative DNA damage and Oct3/4 expression in urinary bladder cancer with *Schistosoma haematobium* infection. *Biochemical and Biophysical Research Communications*.

[15] Thanan R., Pairojkul C., Pinlaor S. (2013). Inflammation-related DNA damage and expression of CD133 and Oct3/4 in cholangiocarcinoma patients with poor prognosis. *Free Radical Biology and Medicine*.

[16] Barbera M., Fitzgerald R. C. (2009). Cellular mechanisms of Bsarrett's esophagus development. *Surgical Oncology Clinics of North America*.

[17] Wild C. P., Hardie L. J. (2003). Reflux, Barrett's oesophagus and adenocarcinoma: burning questions. *Nature Reviews Cancer*.

[18] Hutchinson L., Stenstrom B., Chen D. (2011). Human Barrett's adenocarcinoma of the esophagus, associated myofibroblasts, and endothelium can arise from bone marrow-derived cells after allogeneic stem cell transplant. *Stem Cells and Development*.

[19] Feng H.-L., Liu Y.-Q., Yang L.-J. (2010). Expression of CD133 correlates with differentiation of human colon cancer cells. *Cancer Biology and Therapy*.

[20] Immervoll H., Hoem D., Sakariassen P., Steffensen O. J., Molven A. (2008). Expression of the ‘stem cell marker’ CD133 in pancreas and pancreatic ductal adenocarcinomas. *BMC Cancer*.

[21] Ishigami S., Ueno S., Arigami T. (2010). Prognostic impact of CD133 expression in gastric carcinoma. *Anticancer Research*.

[22] Smith L. M., Nesterova A., Ryan M. C. (2008). CD133/prominin-1 is a potential therapeutic target for antibody-drug conjugates in hepatocellular and gastric cancers. *British Journal of Cancer*.

[23] Wang Q., Chen Z.-G., Du C.-Z., Wang H.-W., Yan L., Gu J. (2009). Cancer stem cell marker CD133+ tumour cells and clinical outcome in rectal cancer. *Histopathology*.

[24] Ahmad J., Arthur K., Maxwell P. (2015). A cross sectional study of p504s, CD133, and Twist expression in the esophageal metaplasia dysplasia adenocarcinoma sequence. *Diseases of the Esophagus*.

[25] Pinlaor S., Hiraku Y., Ma N. (2004). Mechanism of NO-mediated oxidative and nitrative DNA damage in hamsters infected with *Opisthorchis viverrini*: a model of inflammation-mediated carcinogenesis. *Nitric Oxide*.

[26] Hiraku Y., Kawanishi S. (2009). Immunohistochemical analysis of 8-nitroguanine, a nitrative DNA lesion, in relation to inflammation-associated carcinogenesis. *Methods in Molecular Biology*.

[27] Thanan R., Murata M., Ma N. (2012). Nuclear localization of COX-2 in relation to the expression of stemness markers in urinary bladder cancer. *Mediators of Inflammation*.

[28] Murata M., Thanan R., Ma N., Kawanishi S. (2012). Role of nitrative and oxidative DNA damage in inflammation-related carcinogenesis. *Journal of Biomedicine and Biotechnology*.

[29] Thanan R., Oikawa S., Hiraku Y. (2015). Oxidative stress and its significant roles in neurodegenerative diseases and cancer. *International Journal of Molecular Sciences*.

[30] Pinlaor S., Yongvanit P., Hiraku Y. (2003). 8-Nitroguanine formation in the liver of hamsters infected with *Opisthorchis viverrini*. *Biochemical and Biophysical Research Communications*.

[31] Thanan R., Murata M., Pinlaor S. (2008). Urinary 8-oxo-7,8-dihydro-2′-deoxyguanosine in patients with parasite infection and effect of antiparasitic drug in relation to cholangiocarcinogenesis. *Cancer Epidemiology Biomarkers and Prevention*.

[32] Saichua P., Yakovleva A., Kamamia C. (2015). Levels of 8-OxodG predict hepatobiliary pathology in *Opisthorchis viverrini* endemic settings in Thailand. *PLoS Neglected Tropical Diseases*.

[33] Okamoto H., Fujishima F., Nakamura Y. (2013). Significance of CD133 expression in esophageal squamous cell carcinoma. *World Journal of Surgical Oncology*.

[34] Tomizawa Y., Wu T.-T., Wang K. K. (2012). Epithelial mesenchymal transition and cancer stem cells in esophageal adenocarcinoma originating from barrett's esophagus. *Oncology Letters*.

[35] Li W., Tamamura R., Wang B. (2014). Expressions of ABCG2, CD133, and podoplanin in salivary adenoid cystic carcinoma. *BioMed Research International*.

[36] Fukamachi H., Shimada S., Ito K., Ito Y., Yuasa Y. (2011). CD133 is a marker of gland-forming cells in gastric tumors and Sox17 is involved in its regulation. *Cancer Science*.

[37] Hashimoto K., Aoyagi K., Isobe T., Kouhuji K., Shirouzu K. (2014). Expression of CD133 in the cytoplasm is associated with cancer progression and poor prognosis in gastric cancer. *Gastric Cancer*.

[38] Li Y. M., Guo Y. S., Ma B. (2015). CD133 overexpression correlates with clinicopathological features of gastric cancer patients and its impact on survival: a systematic review and meta-analysis. *Oncotarget*.

[39] Sasaki A., Kamiyama T., Yokoo H. (2010). Cytoplasmic expression of CD133 is an important risk factor for overall survival in hepatocellular carcinoma. *Oncology Reports*.

[40] Huang M., Zhu H., Feng J., Ni S., Huang J. (2015). High CD133 expression in the nucleus and cytoplasm predicts poor prognosis in non-small cell lung cancer. *Disease Markers*.

[41] Ohnishi S., Ma N., Thanan R. (2013). DNA damage in inflammation-related carcinogenesis and cancer stem cells. *Oxidative Medicine and Cellular Longevity*.

