# Supplementary figures and images for: DNA Damage in CD133-Positive Cells in Barrett's Esophagus and Esophageal Adenocarcinoma
Source: Mediators Inflamm. 2016 Mar 10;2016:7937814. doi: 10.1155/2016/7937814 (PMC4812016; doi:10.1155/2016/7937814)

## Slide 1
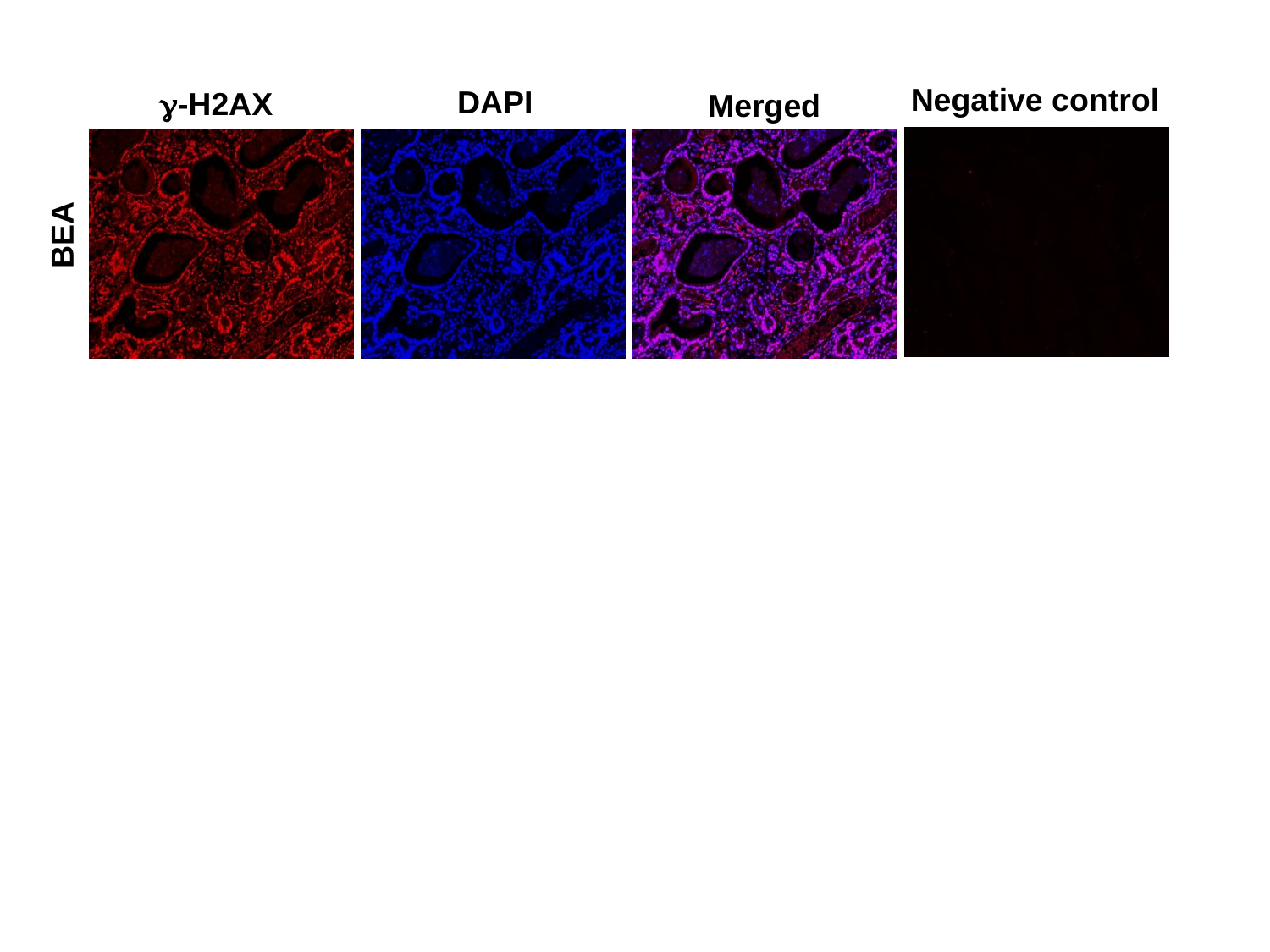

Negative control
DAPI
γ-H2AX
Merged
BEA

Supplement: Supplementary file 1 — Immunohistochemical study of phosphorylated H2AX: Phosphorylated H2AX (γ-H2AX) was stained by using mouse monoclonal anti-phospho-H2AX antibody (clone JBW301, 4 μg/mL, Merck Milipore, Darmstadt, Germany) as a primary antibody. Paraffin sections were incubated with the primary antibodies overnight at room temperature. The sections were next incubated with fluorescent secondary antibody (1:400 Alexa 488-labeled goat anti-mouse IgG (Molecular Probes Inc., Eugene, Oregon, USA) for 3 h at room temperature. Finally, the nuclei were stained by 4'-6-diamidino-2-phenylindole (DAPI) and the sections were examined with a fluorescence microscope (LX70, Olympus, Tokyo, Japan) ora laser scanning confocal microscope (Fluoview FV1000-D, Olympus). For the negative control, we omitted the primary antibody and treated with the secondary antibody according to the procedure. [file 7937814.f1.pptx]
